# Age-Group Differences in BIA-Derived Body Composition and Device-Generated Visceral Fat Rating in a Clinical Cohort

**DOI:** 10.3390/jcm15155814

**Published:** 2026-07-25

**Authors:** Paulina Turko, Bogusław Antoszewski, Marta Fijałkowska

**Affiliations:** Plastic, Reconstructive and Aesthetic Surgery Clinic, Institute of Surgery, Medical University of Lodz, 90-419 Lodz, Poland

**Keywords:** body composition, body mass index, visceral fat rating, bioelectrical impedance analysis

## Abstract

**Background/Objectives:** Body mass index (BMI) is widely used to classify weight status, but it does not reflect fat distribution, muscle mass, or bone mass. Bioelectrical impedance analysis (BIA)-derived visceral fat rating (VFR) is a device-generated estimate of visceral adiposity. This study assessed age- and gender-related differences in anthropometric and BIA-derived body composition parameters, with particular attention to the relationship between BMI classification and VFR. **Methods**: This cross-sectional study included consecutive adults admitted to a single plastic surgery clinic over three months. Waist and hip circumferences were measured, waist-to-hip ratio was calculated, and body composition was assessed using a Tanita BC-420 MA analyzer. Associations between age, BMI, and BIA-derived parameters were assessed using Spearman’s rank correlation. BMI categories were cross-tabulated with VFR categories, and multivariable linear regression was used to assess VFR in relation to age, BMI, and gender. **Results**: The study included 326 patients. Mean BMI was higher in men than in women (27.6 ± 4.33 vs. 26.4 ± 5.07; *p* = 0.006), and men also had higher VFR (12.8 ± 6.15 vs. 7.75 ± 3.74; *p* < 0.001). Older age groups had higher VFR values, ranging from 2.16 ± 1.82 in participants aged 18–29 years to 14.3 ± 5.0 in patients aged ≥ 80 years. BMI and VFR were strongly correlated, but BMI categories did not fully correspond to VFR categories. Manufacturer-defined elevated VFR was observed in 5 patients with normal BMI and 24 patients with overweight, whereas 26 patients classified as obese had a manufacturer-defined normal VFR category. **Conclusions**: In this surgical cohort, older age groups had markedly higher VFR values than younger groups despite relatively modest differences in BMI. Because VFR is a device-generated estimate rather than a direct measure of visceral adiposity, these cross-sectional findings should be interpreted cautiously. Validation against established reference methods is needed before drawing broader clinical conclusions.

## 1. Introduction

Body mass index (BMI) is calculated as the quotient of body weight [kg] and height squared [m^2^]. It was first developed in the 19th century by the Belgian mathematician Adolphe Quetelet, and the term ‘body mass index’ was introduced in 1972 [1]. BMI is a commonly used anthropometric measurement to determine nutritional status. It allows subjects to be classified into four main categories: underweight (BMI < 18.5 kg/m^2^), normal BMI (BMI 18.5–24.9 kg/ m^2^), overweight (25–29.9 kg/m^2^) and obesity (BMI ≥ 30 kg/ m^2^). The last group can be further divided into class I (BMI 30–34.9 kg/ m^2^), II (35–39.9 kg/ m^2^) and III (BMI ≥ 40 kg/ m^2^) obesity.

However, while BMI is a simple and quick measurement, it has important limitations as a stand-alone indicator of adiposity and body composition. It does not reflect age, gender or ethnicity, and it does not provide information on the distribution of fat tissue, muscle mass or bone mass, resulting in an incomplete evaluation of excess body mass [2,3,4,5,6,7]. This may lead to incorrect classification of subjects with increased muscle mass, such as athletes, as overweight or obese. For this reason, BMI should be considered a useful screening tool rather than a complete stand-alone indicator of body composition.

Obesity is one of the most widespread health problems and is associated with an increased risk of cardiometabolic diseases and several cancers, including colorectal and breast cancer [8,9]. However, excessive body mass per se appears to be less informative than the distribution of adipose tissue. It has been found that fat accumulation in the abdominal area, particularly visceral adipose tissue (VAT), is associated with a higher risk of metabolic diseases, some cancers and mortality. In clinical practice, bioelectrical impedance analysis may provide an indirect estimate of visceral adiposity through BIA-generated parameters such as visceral fat rating (VFR) [10,11,12,13]. Fat accumulation in the lower parts of the body, such as the legs, appears to be less strongly associated with adverse cardiometabolic risk than visceral fat accumulation [11].

Body composition may also be assessed using non-invasive bioelectrical impedance analysis (BIA). This method is based on the principle that the volume of a conductor, i.e., water in the human body, is proportional to its length and inversely proportional to its electrical resistance [14,15]. Combined with other information about the individual, such as height, weight and gender, electrical impedance measurement allows estimation of body composition, such as body fat mass (BFM) and fat-free mass (FFM). BFM consists of subcutaneous fat, located mostly in lower parts of the body under the skin and the visceral fat located in the abdomen.

Although BIA is simple, rapid and non-invasive, it remains an indirect method of body composition assessment. Its estimates may be influenced by hydration status, recent food intake, physical activity, time of measurement and device-specific algorithms. Therefore, BIA-derived parameters should be interpreted as estimates of body composition and adiposity distribution, particularly when compared with imaging methods such as dual-energy X-ray absorptiometry, computed tomography or magnetic resonance imaging.

Accurate assessment of nutritional status is particularly important for older people, in whom a normal BMI may mask unfavorable differences in body composition. With age, subcutaneous fat is often redistributed from the lower part of the body to the subcutaneous tissue of the abdominal region and internal organs; in addition, body fat mass increases, while lean mass and bone mineral density decrease [16,17,18]. These differences occur regardless of variations in total fat content, body weight or waist circumference [17]. Many of the age-associated differences in body composition cannot be detected by simple anthropometric measures alone, therefore relying only on BMI to assess the nutritional status of older people may overlook an unfavorable body composition profile.

In this study, we analyzed consecutive patients assessed under routine clinical conditions. BMI remains a useful initial measure of weight status, but it does not describe fat distribution. VFR provides an estimate of visceral adiposity, whereas WHR provides a simple anthropometric descriptor of body fat distribution. Examining these measures together may help determine whether BMI classification reflects BIA-derived visceral adiposity in everyday patient assessment.

Therefore, the aim of this study was to assess differences in anthropometric and BIA-derived body composition parameters across age groups and between genders in a consecutive cohort of adult patients treated in a plastic, reconstructive, and aesthetic surgery clinic. The study also aimed to evaluate the relationship between BMI classification and BIA-derived visceral fat rating.

## 2. Materials and Methods

### 2.1. Study Design and Participants

Consecutive adult patients admitted to the Clinic of Plastic, Reconstructive and Aesthetic Surgery from the beginning of September 2024 to the end of November 2024 were enrolled in this single-center, cross-sectional study. Waist and hip circumferences were measured in all patients, WHR was calculated, and body composition was also analyzed. Each participant underwent a single anthropometric and body composition assessment during the study period. No repeated measurements were performed. Patients were admitted for a broad range of plastic, reconstructive and aesthetic surgical conditions, including skin and soft tissue lesions, post-traumatic and post-oncological defects, congenital deformities, breast conditions, and aesthetic indications.

The inclusion criteria were:

- Age ≥ 18 years;
- Admission to the Clinic during the study period;
- Complete anthropometric and BIA-derived measurements;
- Written informed consent.

The exclusion criteria were:

- Lack of consent;
- Incomplete anthropometric or BIA data;
- Inability to complete the standing BIA measurement.

### 2.2. Anthropometric and Body Composition Measurements

The body composition examination was performed using a body composition analyzer (Tanita BC-420 MA; Tanita Corporation, Tokyo, Japan). The equipment was always located in the same place, in the nurses’ preparation room, and was properly leveled. The patients were asked to remove metal items, such as belts, earrings, and necklaces, as well as their outerwear. Examinations were conducted with patients standing barefoot, with their feet placed on the metal platforms according to the manufacturer’s instructions. Body type, gender, age, and height were entered into the analyzer by the physician before measurement. The results were printed directly and included in the patients’ files.

The Tanita BC-420 MA does not directly measure visceral adipose tissue. Instead, it provides an analyzer-generated estimate of visceral fat rating (VFR) based on bioelectrical impedance analysis together with age, sex, height, and body weight. The mathematical algorithm used to calculate VFR has not been publicly disclosed. According to the manufacturer, one VFR unit corresponds approximately to 10 cm^2^ of MRI-derived visceral fat area [19,20].

Measurements were performed under routine clinical conditions; therefore, fasting status, hydration, physical activity, and time of day were not strictly standardized. This was considered when interpreting BIA-derived parameters. Visceral fat rating and degree of obesity were device-generated parameters obtained directly from the Tanita analyzer. For the exploratory cross-tabulation, VFR values were categorized according to the device output: values of 1–12 were classified as manufacturer-defined normal, whereas values of 13–59 were classified as manufacturer-defined elevated VFR.

Waist circumference was measured in centimeters; the measurement was taken by tape-measure exactly halfway between the lower edge of the costal arch and the upper iliac crest. Hip circumference was measured with the same tape at the height of the anterior superior iliac spines or the widest part of the buttocks. All measurements were performed by the same doctor.

BMI was calculated as body weight divided by height squared (kg/m^2^), and weight status categories were assigned according to standard BMI cut-off values: underweight (BMI < 18.5 kg/m^2^), normal weight (BMI 18.5–24.9 kg/m^2^), overweight (BMI 25–29.9 kg/m^2^), and obesity (BMI ≥ 30 kg/m^2^).

### 2.3. Ethical Considerations

All patients signed written consent forms prior to enrollment in the study. The project was approved by the Ethics Review Board of the Medical University of Lodz (approval no. RNN/223/23/KE and RNN/77/24/KE), and the study was conducted following the principles of the Declaration of Helsinki.

### 2.4. Statistical Analysis

Statistical analysis was performed using Statistica 13. Continuous variables are presented as mean ± standard deviation for descriptive purposes. Because the distributions of the analyzed continuous variables deviated from normality (Shapiro–Wilk test), the Mann–Whitney U test was used for comparisons between two groups and the Kruskal–Wallis test for comparisons across more than two groups. Spearman’s rank correlation coefficient was calculated to assess associations between age, BMI, and BIA-derived parameters. All group comparisons and correlation analyses were unadjusted and should be interpreted as descriptive analyses. Adjustment for potential confounders was performed only in the multivariable regression model.

To explore the relationship between BMI-based weight status and BIA-derived visceral adiposity, BMI categories were cross-tabulated with VFR categories, and Pearson’s chi-square test was used to assess the association between these categorical variables.

An exploratory multivariable linear regression model was constructed to assess the association between age and VFR after adjustment for BMI and gender. VFR was entered as the dependent variable, while age, BMI, and gender were entered as independent variables. Gender was coded as female = 0 and male = 1 for regression analyses. Statistical significance was set at *p* < 0.05. Model diagnostics included assessment of multicollinearity (tolerance and variance inflation factor), residual normality, and residual plots.

## 3. Results

### 3.1. Characteristics of the Study Population

A total of 354 consecutive adult patients were assessed for eligibility. Sixteen patients declined participation, two were excluded because standing BIA measurement could not be performed (one forefoot amputation and one wheelchair user), and ten were excluded because of incomplete anthropometric or body composition measurements. Consequently, 326 participants were included in the final analyses. No missing data were present in the final analytical dataset because participants with incomplete anthropometric or body composition measurements were excluded before analysis. Of the 326 participants, 228 were female (69.9%) and 98 were male (30.1%). The participants were divided into seven groups according to age: 18–29 years (38 participants; 11.7%), 30–39 (17; 5.2%), 40–49 (45; 13.8%), 50–59 (63; 19.3%), 60–69 (65; 19.9%), 70–79 (67; 20.6%), and ≥80 years (31; 9.5%). Regarding gender, the mean age was 56.6 ± 17.2 years (range 18 to 87 years) for women, and 60.0 ± 19.7 years (range 19 to 92 years) for men. Women were significantly younger than men (*p* = 0.038). The age difference should be considered when interpreting gender-related comparisons.

According to BMI classification, 122 subjects had a normal body weight (37.4%), four were underweight (1.2%), 130 were overweight (39.9%) and the remaining 70 were obese (21.5%). BMI ranged from 18.0 to 47.9 (mean 26.4 ± 5.07) among the women and from 19.2 to 41.0 (mean 27.6 ± 4.33) among the men; the difference was statistically significant (*p* = 0.006). The mean visceral fat rating (VFR) was 7.75 ± 3.74 in women, and 12.8 ± 6.15 in men (*p* < 0.001); the mean degree of obesity was 19.9 ± 23.3% in women, and 24.9 ± 18.9% in men (*p* = 0.005).

The men demonstrated significantly higher muscle mass, i.e., 61.0 ± 7.95 kg vs. 43.2 ± 5.14 kg (*p* < 0.001) and bone mass, i.e., 3.19 ± 0.38 kg vs. 2.31 ± 0.26 kg than the women (*p* < 0.001). In contrast, the women exhibited significantly greater fat mass, i.e., 25.0 ± 10.3 kg vs. 21.4 ± 9.73 kg (*p* < 0.001) (Table 1).

Among the 228 women, 47 had WHR < 0.8 (20.6%) and 181 had WHR ≥ 0.8 (79.4%); the mean WHR was 0.86 ± 0.09, ranging from 0.68 to 1.21. Among the men, 68 had WHR < 1 (69.4%), and 30 with WHR ≥ 1 (30.6%); the mean value was 0.96 ± 0.09, ranging from 0.78 to 1.19 (Table 1).

### 3.2. Relationship Between BMI Categories and Visceral Fat Rating

To explore the relationship between BMI-based weight status and BIA-derived visceral adiposity, BMI categories were cross-tabulated with manufacturer-defined VFR categories. VFR values of 1–12 were classified as normal, whereas values of 13–59 were classified as elevated according to the device output. BMI category was significantly associated with VFR category (Pearson’s chi-square test: χ^2^ (3, *n* = 326) = 91.76, *p* < 0.001). However, BMI and VFR categories were not fully concordant. manufacturer-defined elevated VFR was observed in 5 patients with normal BMI (4%) and in 24 patients with overweight (18%). Conversely, 26 patients classified as obese according to BMI had a manufacturer-defined normal VFR category (37%) (Table 2).

### 3.3. Age-Related Differences and Associations in Body Composition Parameters

BMI differed significantly across age groups (*p* < 0.001). The youngest age group (18–29 years) had the lowest mean BMI (22.8 ± 3.44 kg/m^2^), whereas all other groups had mean BMI values within the overweight range (Table 3). Although BMI was generally higher in older age groups, participants aged 70–79 years had a lower mean BMI than those aged 60–69 years and ≥80 years. BMI showed a modest positive correlation with age (rho = 0.30, *p* < 0.001).

An interesting relationship was observed between age and fat mass and degree of obesity. Older subjects tended to have more fat mass and a significantly higher degree of obesity; however, the fat mass, and hence degree of obesity, was found to fall after the age of 70. In respondents over 80 years old, fat mass remained approximately at the same level as in a decade earlier, while BMI and degree of obesity increased (Table 3). Spearman’s rank correlation coefficient for the associations with age were 0.25 for fat mass and 0.30 for degree of obesity (*p* < 0.001 for both). These findings indicate significant but relatively weak positive associations between age and these parameters.

**Figure 1:**
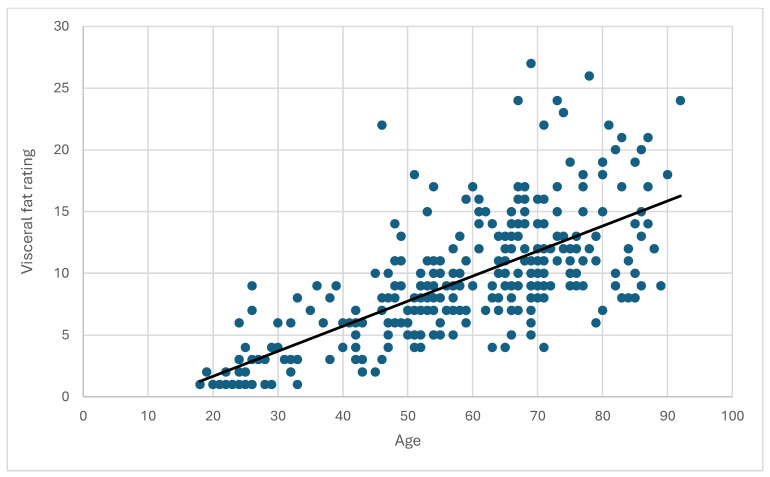
Association between age and BIA-derived visceral fat rating in the study cohort, assessed using Spearman’s rank correlation.

While muscle mass and bone mass appeared to differ across age groups, these variations were not statistically significant (*p* > 0.05). However, older age groups had progressively higher VFR values, ranging from 2.16 ± 1.82 in participants aged 18–29 years to 14.3 ± 5.0 in participants aged ≥ 80 years (Table 3). VFR demonstrated a strong relationship with age (Spearman’s rho = 0.73, *p* < 0.001) (Figure 1).

### 3.4. Exploratory Multivariable Analysis of Factors Associated with Device-Generated VFR

BMI was strongly positively correlated with VFR (Spearman’s rho = 0.73, *p* < 0.001). This indicates that BMI and VFR were related, although the cross-tabulation showed that their categorical interpretation was not identical in all patients.

An exploratory multivariable linear regression analysis was performed with VFR as the dependent variable and age, BMI, and gender as independent variables. The model was statistically significant and explained 89.9% of the variance in VFR (R^2^ = 0.899, adjusted R^2^ = 0.898, F(3.322) = 954.34, *p* < 0.001). Detailed regression coefficients are presented in Table 4. Because age and gender contribute to the algorithm used to derive VFR, these findings should be interpreted as descriptive and exploratory rather than as evidence of independent biological associations.

Tolerance values ranged from 0.899 to 0.984 (corresponding VIF values 1.02–1.11), indicating no evidence of problematic multicollinearity. Residual plots showed no major violations of model assumptions.

## 4. Discussion

The present study showed that, in this single-center surgical cohort, older age groups had markedly higher BIA-derived VFR values than younger participants, and that VFR showed a stronger cross-sectional association with age than BMI. BMI and VFR were strongly correlated, although their categorical classifications were not fully concordant. Age remained statistically associated with VFR after adjustment for BMI and gender in the exploratory regression model. However, because age contributes directly to the algorithm used to derive VFR, this association should not be interpreted as evidence of an independent biological relationship. The present study did not evaluate BMI against a reference method or clinical outcomes; therefore, the findings should be interpreted as a descriptive comparison rather than a validation study.

Despite being used for almost 200 years, BMI does not seem to be a perfect indicator of a patient’s health status or body composition. Indeed, Lebiedowska et al. propose that it should rather serve as a tool for pre-estimating abnormal weight, and body composition analysis should be performed if possible [21]. The authors performed a bioelectrical impedance analysis of 267 healthy women and found that BMI was a highly inaccurate indicator for diagnosing body composition disorders: some women who had normal weight or were even underweight showed high body fat content, while others with high BMI, indicating obesity, had low abdominal fat (favorable distribution of subcutaneous fat within the lower limbs and buttocks). As such, a reliance on BMI can mask various types of body composition irregularities, and body composition appears to be a more detailed form of analysis.

The present results further support this observation by showing that BMI-based weight status and BIA-derived VFR categories were related but not fully concordant. Although BMI category was significantly associated with VFR category, discrepancies between the two classifications were observed. Manufacturer-defined elevated VFR was found in some patients with normal BMI or overweight, whereas some patients classified as obese according to BMI had a manufacturer-defined normal VFR category. This does not undermine the role of BMI as an initial screening measure but indicates that BMI classification and BIA-derived visceral adiposity reflect related, yet not identical, aspects of body composition.

Furthermore, body composition differs across age groups and is known to change with aging. Taking measurements of only body mass, or even mass and height with BMI calculation, may mask the actual distribution of fat in the patient, and therefore not properly reflect the body composition profile. It has been suggested that body weight can remain stable or even continue to increase until the age of 70 in men and women [22,23]. In our cross-sectional cohort, mean BMI was generally higher in older age groups up to 60–69 years, decreased slightly in the 70–79-year group, and was highest among participants aged ≥ 80 years. However, it appears that body weight then decreases slightly and then increases again after the age of 80; our present findings indicate the highest BMI was 28.69 in this age group.

Similarly, total body fat has also been found to increase with age in men and women until the age of 70 and then decrease; however, in patients after 80 years of age, total body fat remains stable, but BMI and degree of obesity increase [24]. It has been reported that aging is associated with redistribution of fat mass: more specifically, an increase in visceral fat deposition and a decrease in muscle mass. This has important negative clinical implications because it is an independent risk factor for the development of many diseases [12,13,25]. Some results indicate that the circumference of the waist, or of some other parts of the body, is positively related to increased visceral adiposity; as such, a quick measurement can be helpful in estimating abdominal obesity. However, some studies indicate that aging is associated with increased level of visceral fat, independent of waist circumference [26]. Our findings showed that older age groups had progressively higher estimated VFR values, reaching a mean value of 14.3 in participants aged ≥ 80 years. This observation should be interpreted cautiously because VFR is an analyzer-generated estimate and age contributes to the underlying algorithm used to derive the VFR score.

The exploratory regression model supported the descriptive observation that higher age groups tended to have higher VFR values after adjustment for BMI and gender. However, because age and gender are incorporated into the VFR algorithm, these findings should not be interpreted as evidence of independent biological associations.

However, patients with the same waist circumference but different height will be at different risks of developing cardiovascular diseases or metabolic disturbances [27]. While abdominal obesity is recognized as a key risk factor in these disorders, it is important to differentiate between two types of abdominal fat distribution. Although the masses of both visceral and subcutaneous adipose tissue compartments are correlated with a greater risk of metabolic disorders, visceral fat remains more strongly associated with an adverse metabolic risk profile [28]. Visceral adiposity is recognized as an important component of body composition assessment. Body circumference and BMI remain useful screening measures but may be interpreted alongside measures that provide information on body fat distribution. Our data indicate that older age groups had progressively higher BIA-derived VFR values, ranging from 2.16 in patients aged 18–29 years to 14.3 in participants aged ≥ 80 years. Nevertheless, the present study did not include metabolic outcomes; therefore, no direct conclusions regarding metabolic risk can be drawn from these data.

These findings should be considered in relation to other methods of body composition assessment. BMI remains useful for classifying weight status, but it does not measure regional adiposity. Visceral adipose tissue is more closely associated with metabolic risk than general body size alone [29]. For this reason, methods that describe fat distribution are clinically relevant when body composition is assessed beyond BMI. CT and MRI provide a more direct assessment of visceral adipose tissue, whereas DXA allows detailed evaluation of total and regional body composition [29,30].

Studies comparing BIA with imaging-based methods show why BIA-derived VFR should be interpreted as a device-generated estimate rather than a direct measurement of visceral fat. Xu et al. compared single-frequency BIA with CT and found only limited agreement between BIA-derived and CT-derived visceral fat measurements [31]. Pietiläinen et al. compared BIA with DXA and MRI during a 12-month weight loss intervention and showed that the agreement depended on the body composition component assessed. BIA correlated strongly with DXA for fat mass, but visceral fat estimation was less consistent when compared with MRI; BIA tended to overestimate visceral fat, particularly at higher visceral fat levels and especially in men [32].

The present study was not designed to validate BIA-derived VFR against imaging methods. Rather, it showed that BMI classification and VFR categories were associated but not completely concordant in routine clinical practice.

Therefore, the estimated VFR should not be regarded as a substitute for imaging-based assessment of visceral adipose tissue. Its value in this setting is different: when obtained as part of routine body composition analysis, it may provide descriptive information on visceral adiposity without radiation exposure or additional imaging procedures. The present findings indicate that BMI classification and VFR categories are associated but not fully concordant in this cohort.

### 4.1. Limitations of the Study

Several limitations should be acknowledged. First, this was a cross-sectional, single-center study, and no longitudinal observations were performed; therefore, the results should be interpreted as age-related differences across groups rather than individual changes over time. Second, the study population consisted of patients treated in a plastic, reconstructive and aesthetic surgery clinic, which may limit the generalizability of the findings to broader clinical or community-based populations. Third, BIA is an indirect method of body composition assessment and may be influenced by hydration status, recent food intake, physical activity, edema, and time of measurement and device-specific algorithms. In the present study, measurements were performed under routine clinical conditions rather than according to a fully standardized fasting protocol. Fourth, multiple comparisons were performed without correction for multiple testing; therefore, individual *p*-values from exploratory group comparisons should be interpreted cautiously. Fifth, no reference method such as dual-energy X-ray absorptiometry, computed tomography or magnetic resonance imaging was used to validate BIA-derived parameters. Therefore, VFR should be interpreted as a device-generated estimate rather than a direct measurement of visceral adipose tissue. In addition, because age and gender contribute to the algorithm used to derive VFR, the regression findings should be interpreted as exploratory.

### 4.2. Recommendations for Future Research

Future studies should validate BIA-derived VFR against reference or imaging-based methods, including DXA, CT, or MRI, using standardized measurement protocols. Prospective and multicenter studies would help determine whether the observed relationship between BMI classification and BIA-derived visceral adiposity is consistent across different clinical and community-based populations. Future research should also include metabolic outcomes, such as glucose metabolism, lipid profile, blood pressure, and cardiovascular risk markers, to clarify the clinical significance of discordance between BMI categories and VFR categories. Longitudinal studies are needed to determine whether changes in VFR over time provide additional information beyond BMI in the assessment of body composition and metabolic risk.

## 5. Conclusions

In this cross-sectional surgical cohort, BMI categories and BIA-derived VFR categories were strongly associated but not fully concordant. Older age groups had higher VFR values than younger groups. Because VFR is a device-generated estimate rather than a direct measure of visceral adiposity, these findings should be interpreted cautiously. Further validation against imaging-based reference methods and clinical outcomes is required before conclusions regarding its diagnostic accuracy or clinical utility can be drawn.

## Figures and Tables

**Table 1:** Comparison of anthropometric and BIA-derived body composition parameters between women and men in the study cohort.

Variables	Gender				Z Test Value	*p*
	Men		Women			
	Mean	SD	Mean	SD		
Age	60.0	19.7	56.6	17.2	2.078	0.038
WHR	0.96	0.089	0.86	0.089	-	-
BMI	27.6	4.33	26.4	5.07	2.766	0.006
% fat	24.0	7.54	34.0	8.19	9.061	<0.001
Fat mass	21.4	9.73	25.0	10.3	3.067	0.002
Muscle mass	61.0	7.95	43.2	5.14	13.433	<0.001
Bone mass	3.19	0.38	2.31	0.26	13.413	<0.001
TBW %	52.3	5.13	46.0	5.65	9.019	<0.001
% degree of obesity	24.9	18.9	19.9	23.3	2.786	0.005
visceral fat rating	12.8	6.15	7.75	3.74	7.702	<0.001

Values are presented as mean and standard deviation. Differences between women and men were assessed using the Mann–Whitney U test; Z values are reported. WHR was presented descriptively because its interpretation relies on gender-specific cut-off values. BMI, body mass index; WHR, waist-to-hip ratio; TBW, total body water.

**Table 2:** Distribution of BIA-derived visceral fat rating categories across BMI categories in the study cohort.

BMI Category	Manufacturer-Defined Normal VFR, *n* (%)	Manufacturer-Defined Elevated VFR, *n* (%)	Total, *n*
Underweight	4 (100%)	0 (0%)	4
Normal weight	117 (96%)	5 (4%)	122
Overweight	106 (82%)	24 (18%)	130
Obesity	26 (37%)	44 (63%)	70
Total	253 (78%)	73 (22%)	326

Values are presented as *n* and row percentage. VFR values of 1–12 were classified as manufacturer-defined normal and values of 13–59 as manufacturer-defined elevated according to the device output. The association between BMI category and VFR category was assessed using Pearson’s chi-square test. BMI, body mass index; VFR, visceral fat rating.

**Table 3:** Comparison of anthropometric and BIA-derived body composition parameters across age groups in the study cohort.

Variables	Age (Years)														H Test Value	*p*
	18–29		30–39		40–49		50–59		60–69		70–79		≥80			
	Mean	SD	Mean	SD	Mean	SD	Mean	SD	Mean	SD	Mean	SD	Mean	SD		
BMI	22.8	3.44	25.9	3.51	26.4	5.55	26.8	3.47	28.1	5.69	27.4	3.96	28.7	5.91	42.44	<0.001
% fat	21.2	9.04	27.9	9.71	31.8	8.19	33.1	7.62	33.1	9.61	32.6	7.67	31.9	8.36	46.42	<0.001
Fat mass	14.5	8.54	21.8	9.27	24.8	11.2	25.0	7.67	26.9	11.6	24.8	8.27	24.8	10.8	47.60	<0.001
Muscle mass	49.5	9.85	52.0	12.4	47.9	11.1	47.8	10.7	49.4	10.4	47.4	8.96	48.3	9.58	5.460	0.486
Bone mass	2.61	0.49	2.78	0.61	2.56	0.53	2.53	0.52	2.63	0.52	2.51	0.45	2.56	0.47	5.812	0.445
TBW %	55.2	5.93	50.3	5.91	47.8	4.80	46.9	6.13	46.2	5.84	46.4	5.04	46.6	5.60	58.30	<0.001
% degree of obesity	3.83	15.7	16.0	17.2	20.1	25.3	21.7	16.5	27.0	16.2	23.6	16.5	30.4	26.8	41.44	<0.001
Visceral fat rating	2.16	1.82	5.00	2.55	7.00	3.77	8.51	2.92	11.3	4.27	12.4	4.13	14.3	4.97	176.5	<0.001

Values are presented as mean and standard deviation. Comparisons between age groups were performed using the Kruskal–Wallis test; H values are reported. BMI, body mass index; TBW, total body water.

**Table 4:** Results of the exploratory multivariable linear regression analysis assessing the association of age, BMI, and gender with device-generated visceral fat rating (VFR).

Predictor	B (95% CI)	SE	Standardized β	*p*
Age (years)	0.149 (0.139–0.160)	0.005	0.522	<0.001
BMI (kg/m^2^)	0.544 (0.505–0.582)	0.020	0.516	<0.001
Gender	3.931 (3.536–4.325)	0.200	0.350	<0.001

Dependent variable: device-generated visceral fat rating (VFR). Regression coefficients (B), standard errors (SE), standardized regression coefficients (β), and 95% confidence intervals (CI) are presented. The model explained 89.9% of the variance in VFR (R^2^ = 0.899; adjusted R^2^ = 0.898; F(3.322) = 954.34; *p* < 0.001). Gender was coded as female = 0 and male = 1.

## Data Availability

The data that support the findings of this study are available from the corresponding author upon reasonable request.

## Abbreviations

The following abbreviations are used in this manuscript:

BMI	Body Mass Index
BIA	Bioelectrical impedance analysis
WHR	Waist-to-hip ratio
VFR	Visceral fat rating
VAT	Visceral adipose tissue
VFA	Visceral fat area
